# No evidence for Peto’s paradox in terrestrial vertebrates

**DOI:** 10.1073/pnas.2422861122

**Published:** 2025-02-24

**Authors:** George Butler, Joanna Baker, Sarah R. Amend, Kenneth J. Pienta, Chris Venditti

**Affiliations:** ^a^University College London Cancer Institute, University College London, London WC1E 6DD, United Kingdom; ^b^Cancer Ecology Center, The Brady Urological Institute, Johns Hopkins School of Medicine, Baltimore, MD 21287; ^c^School of Biological Sciences, University of Reading, Reading RG6 6AS, United Kingdom

**Keywords:** Peto’s paradox, cancer evolution, comparative phylogenetics

## Abstract

Larger, longer-lived species are expected to have a higher cancer prevalence compared to smaller, shorter-lived species owing to the greater number of cell divisions that occur during their lifespan. Yet, to date, no evidence has been found to support this expectation, and no association has been found between cancer prevalence and body size across species—a phenomenon known as Peto’s paradox. Specifically, while anticancer mechanisms have been identified for individual species, wider phylogenetic evidence has remained elusive. Here, we show that there is no evidence for Peto’s paradox across amphibians, birds, mammals, and squamate reptiles: Larger species do in fact have a higher cancer prevalence compared to smaller species. Moreover, we demonstrate that the accumulation of repeated instances of accelerated body size evolution in mammals and birds is associated with a reduction in the prevalence of neoplasia and malignancy, suggesting that increased rates of body size evolution are associated with the evolution of improved cellular growth control. These results represent empirical evidence showing that larger body size is related to higher cancer prevalence, thus rejecting Peto’s paradox, and demonstrating the importance of heterogenous routes of body size evolution in shaping anticancer defenses.

## Significance

Peto’s paradox—the puzzling disconnect between body size and cancer prevalence across species—has long stood as one of comparative biology’s most captivating and unresolved enigmas. Through the application of advanced phylogenetic comparative methods, we reveal that empirical evidence does not support Peto’s paradox across the four major classes of terrestrial vertebrates: Larger species do, in fact, face higher cancer prevalence than their smaller counterparts. Furthermore, we show that as variation in body size has evolved in birds and mammals, it has also driven the evolution of enhanced cellular growth control. This adaptation allows species to grow larger over time without experiencing the anticipated cancer burden associated with their size.

All multicellular species rely upon cell division for tissue growth and repair. However, errors during the division process can lead to somatic mutations that accumulate over time which may potentially underpin the emergence of cancer (malignant tumors that invade benign tissue and metastasize) (1, 2). Thus, all else being equal, larger, longer-lived species are expected to have an increased cancer prevalence compared to smaller, shorter-lived species (3), an expectation that has been shown at the intraspecies level in humans (4) and dogs (5). Yet, despite these predictions, no association has been previously found between cancer prevalence and body size across species, an observation known as Peto’s paradox (6). While species-specific anticancer mechanisms have been identified, for example in extant Proboscideans (elephants) which has a low prevalence of cancer has ~20 copies of the tumor suppressor gene TP53 (7), or *Myotis pilosus* (Rickett’s big-footed bat) which also has a low prevalence of cancer has downregulation of multiple known cancer-associated genes (*HIF1A*, *COPS5*, and *RPS*) (8), yet broader empirical or analytic phylogenetic evidence has remained elusive.

Early attempts to test Peto’s paradox suffered from a lack of statistical power owing to the small number of species sampled and the limited number of necropsies conducted per species (9, 10). However, recent efforts have helped overcome these issues allowing Peto’s paradox to be tested at an unprecedented scale across multiple vertebrate classes (11–13). As a result, a positive association between neoplasia (all tumors both benign and malignant) prevalence and body size has been reported in some vertebrate groups (11, 13). However, no study to date has found a similar association between malignancy and body size: the crux of Peto’s paradox (11–13). Crucially, previous attempts to test Peto’s paradox have relied upon first quantifying the proportion of malignancy with respect to the number of necropsies for each species before then testing for an association with body size (9–11, 13). However, high levels of variation in the amount of malignancy per species will result in an underestimation of the proportion of malignancy relative to body size (14). In contrast, testing Peto’s paradox using the observed number of malignancies directly for each species in a regression framework aims to minimize the total residual error across the entire dataset rather than biasing the estimate toward values close to the mean. Furthermore, proportions do not *evolve* as would be expected in accordance with Brownian motion, the underlying assumption of most phylogenetic comparative models. As a result, the estimated Brownian variance lacks interpretability and biological meaning, an aspect that is avoided by modeling the number of malignancies directly for each species.

Briefly, Brownian motion is commonly used to describe the random evolution of traits through time. Specifically, changes in the value of a trait are drawn from a normal distribution with mean 0 and variance equal to the product of the rate of evolution, σ^2^, and the amount of time that has elapsed, t, (variance = s^2^t). Crucially, the rate of evolution describes how fast the trait is expected to change through time. For instance, in the context of body size, a high rate of evolution means that the change in body size between the ancestor and descendant is large, irrespective of whether the body size of the descendant has increased or decreased with respect to the ancestor. However, a similar biological interpretation is not possible with respect to proportions. That is, the estimated rate of evolution might capture a difference in the number of malignancies between the ancestor and the descendant or the number of necropsies. Given that the number of necropsies does not evolve through time, the rate of evolution no longer holds the same biological meaning and thus violates the underlying assumptions of the model.

Here, we use a Bayesian phylogenetic framework (15) to test Peto’s paradox across a dataset spanning four vertebrate classes (13): amphibians, birds, mammals, and squamate reptiles. If Peto’s paradox is true, we expect to find no association between malignancy and body size after accounting for the number of necropsies per species. In contrast, if Peto’s paradox is false, we expect to find a positive association between malignancy and body size after accounting for the number of necropsies per species.

## Results

### Testing Peto’s Paradox.

Prevalence data, both neoplastic and malignant, are from ref. 13, and the body size data are from ref. 16. The paired body size and prevalence data used throughout contain 263 species across four vertebrate classes: 31 amphibians, 79 birds, 90 mammals, and 63 squamate reptiles (*Materials and Methods*). Owing to the sparsity of body mass information available for amphibians and reptiles, snout–vent length (SVL) was used as a proxy for body mass. SVL is a commonly used proxy for body size in species with indeterminant growth, such as amphibians and squamate reptiles (17). To account for potential differences in growth dynamics, i.e., determinate vs indeterminate growth, we analyzed amphibians and reptiles separately from birds and mammals.

**Fig. 1.**
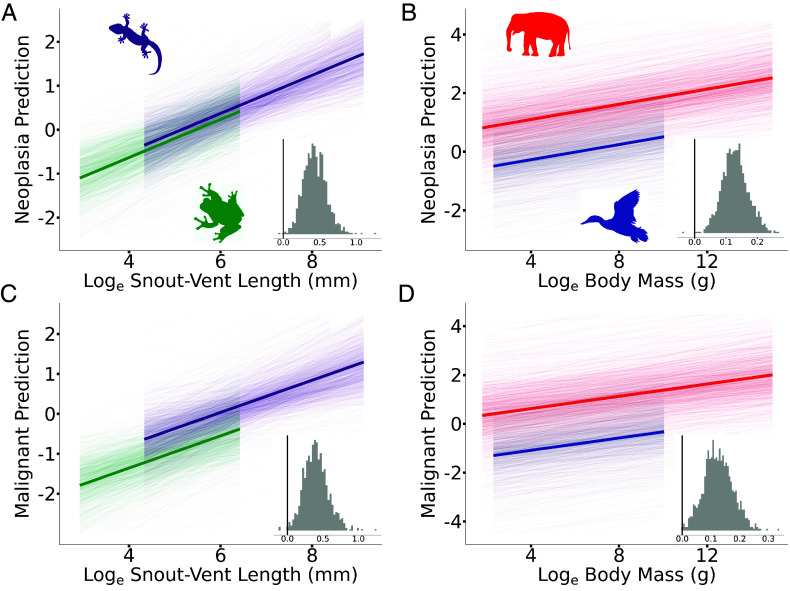
A positive association between neoplasia or malignancy and body size in terrestrial vertebrates. In all cases, the posterior predicted slopes are plotted, and the mean average predicted slopes are highlighted. Insets show the posterior distribution of the estimated slopes, and the black vertical line indicates 0 on the x-axis. A slope is significant if less than 5% of the posterior distribution crosses 0. Neoplasia prevalence is positively associated with (*A*) snout–vent length (SVL) in amphibians (green) and reptiles (purple) (P_x_ = 0.002) and (*B*) body mass in birds (blue) and mammals (red) (P_x_ = 0.001). Malignancy prevalence is positively associated with (*C*) SVL in amphibians and reptiles (P_x_ = 0.003) and (*D*) body mass in birds and mammals (P_x_ = 0.001).

**Fig. 2.**
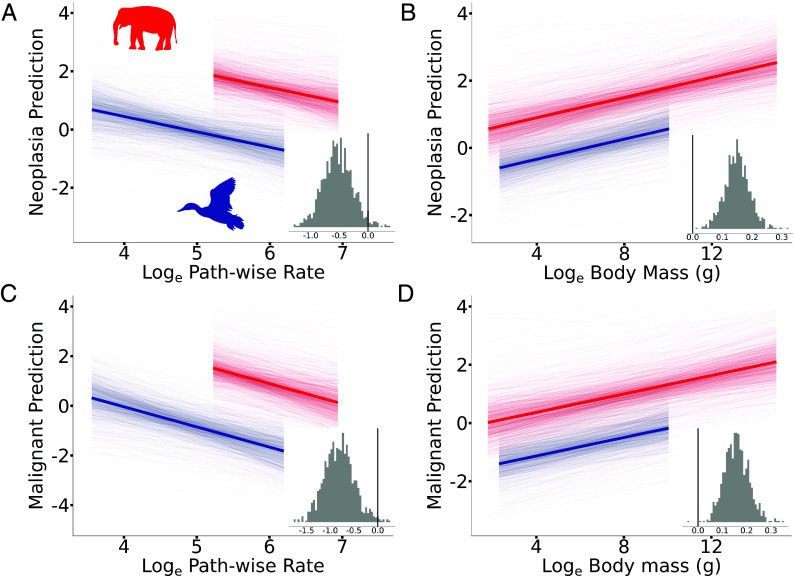
A negative association between neoplasia or malignancy and pathwise rate in birds and mammals. In all cases, the posterior predicted slopes are plotted, and the mean average predicted slopes are highlighted. Insets show the posterior distribution of the estimated slopes, and the black vertical line indicates 0 on the x-axis. A slope is significant if less than 5% of the posterior distribution crosses 0. (*A*) Neoplasia prevalence is negatively associated with pathwise rate (*Materials and Methods*) in birds (blue) and mammals (red) (P_x_ = 0.027) but (*B*) positively associated with body mass in the same model (P_x_ = 0). Likewise, (*C*) malignancy is negatively associated with pathwise rate in birds and mammals (P_x_ = 0.01) but (*D*) positively associated with body mass (P_x_ = 0.001).

To test Peto’s paradox, we used a multivariate phylogenetic generalized linear mixed model (MPGLMM) in a Bayesian framework (*Materials and Methods*) (18). Specifically, in contrast to previous studies (10, 11, 13), we used a Poisson regression to directly model the observed number of neoplasia and malignancies for each species rather than modeling the species-specific proportion. Note that this is not possible in analyses using conventional phylogenetic generalized least squared models as have previously been used. To ensure that our results were not being driven by a small number of potential outlier species, we removed species with a studentized residual error greater than three (*Materials and Methods*). We removed a total of 11 species: two amphibians, two birds, three mammals, and four squamate reptiles. All results are presented with the outlier species removed although we found similar qualitative results with the 11 outlier species included (*SI Appendix*, Figs. S1, S2, S7, and S8). Finally, the y-axis in Figs. 1 and 2 and *SI Appendix*, Figs. S1–5 and S7–S12 is labeled as “Neoplasia prediction” or “Malignancy prediction” to indicate that it is an estimate from the model rather than a sampled value. Put simply, the y-axis can be thought of as the number of neoplasia or malignancy expected for a certain value on the x-axis (e.g., for a given body size or pathwise rate) given the estimated model.

### Evaluating Species Body Size.

To test Peto’s paradox, we fitted an MPGLMM in which both neoplasia and malignancy were dependent on the number of necropsies and body size in a single model. A single slope was estimated for the number of necropsies while separate intercepts and body size slopes were estimated for each class. We found no significant difference between the body size slopes for neoplasia in amphibians and squamate reptiles (P_x|diff_ = 0.138, *SI Appendix*, Fig. S3*A*) or birds and mammals (P_x|diff_ = 0.465, *SI Appendix*, Fig. S3*B*). Likewise, we found no significant difference between the body size slopes for malignancy in amphibians and squamate reptiles (P_x|diff_ = 0.076, *SI Appendix*, Fig. S3*C*) or birds and mammals (P_x|diff_ = 0.344, *SI Appendix*, Fig. S3*D*). As a result, we refitted a single body size slope for amphibians and squamate reptiles and a single slope for birds and mammals, the intercepts remained separate for each class.

We found a significant positive association between neoplasia and SVL in amphibians and squamate reptiles (P_x_ = 0.002, β = 0.433, 95% CI = 0.138 to 0.748, Fig. 1*A*) and a significant positive association between neoplasia and body mass in birds and mammals (P_x_ = 0.001, β = 0.129, 95% CI = 0.052 to 0.215, Fig. 1*B*). Across all four vertebrate classes, larger species have an increased prevalence of neoplasia compared to smaller species. We also found a significant positive association between malignancy and SVL in amphibians and squamate reptiles (P_x_ = 0.003, β = 0.403, 95% CI = 0.086 to 0.756, Fig. 1*C*) and a significant positive association between malignancy and body mass in birds and mammals (P_x_ = 0.001, β = 0.126, 95% CI = 0.029 to 0.221, Fig. 1*D*). Finally, we found that body mass explained a significant proportion of the variation in neoplasia and malignancy in amphibians and reptiles (marginal R^2^ = 0.616 and conditional R^2^ = 0.777; marginal R^2^ refers to the variance explained by all fixed effects and conditional R^2^ to all fixed and random effects together) and birds and mammals (marginal R^2^ = 0.445 and conditional R^2^ = 0.770). Across all four vertebrate classes, larger species have an increased prevalence of malignancy compared to smaller species, thus demonstrating no evidence of Peto’s paradox.

### Investigating Species Longevity and Age at Death.

To ensure that differences in species longevity were not driving our results, we refitted both models (birds and mammals as well as amphibians and reptiles) with longevity included as an independent variable (19) with a single slope estimated for amphibians and squamate reptiles and birds and mammals respectively. Owing to limited longevity data, our dataset for these analyses is reduced slightly, including 12 amphibians, 45 birds, 83 mammals, and 37 squamate reptiles.

We found that species longevity was not a significant predictor of neoplasia in amphibians and squamate reptiles (P_x_ = 0.1, β = 0.592, 95% CI = −0.264 to 1.523, *SI Appendix*, Fig. S4*A*) or birds and mammals (P_x_ = 0.245, β = −0.158, 95% CI = −0.573 to 0.295, *SI Appendix*, Fig. S4*B*). Likewise, species longevity was not a significant predictor of malignancy in amphibians and squamate reptiles (P_x_ = 0.118, β = 0.560, 95% CI = −0.346 to 1.555, *SI Appendix*, Fig. S4*C*) or birds and mammals (P_x_ = 0.21, β = −0.212, 95% CI = −0.777 to 0.337, *SI Appendix*, Fig. S4*D*). Thus, these findings cannot be attributed to species longevity. However, we acknowledge that more expansive longevity data are required to fully elucidate the effect of species longevity.

Similarly, the age at death for the individual animals at the time of necropsy was also tested in a subset of species (13) (*Materials and Methods*). Age at death information was not publicly available for all species in which a necropsy had been conducted, and thus, our dataset for these analyses was reduced, comprising: 1 amphibian, 12 birds, 49 mammals, and 11 squamate reptiles. The limited number of amphibians and squamate reptiles meant that further analysis was not feasible in these classes. In birds and mammals, we fitted a model with the average age at death included as an independent variable with a single slope estimated for both classes. We found that age at death was not a significant predictor of neoplasia (P_x_ = 0.221, β = 0.158, 95% CI = −0.198 to 0.553, *SI Appendix*, Fig. S5*A*) or malignancy (P_x_ = 0.302, β = 0.145, 95% CI = −0.340 to 0.641, *SI Appendix*, Fig. S5*B*).

We had insufficient data to test the age at death for amphibians and squamate reptiles. However, we do not anticipate that the results would be significantly different. For the age at death to be a significant predictive variable, shorter-lived species would need to live multiple times longer compared to longer-lived species. Given that shorter-lived species typically experience a greater relative increase in lifespan when in captivity compared to longer-lived species (20), and the longevity data used in our model were quantified from species in captivity (19), we do not anticipate that the age at death would result in a qualitatively different result from the longevity estimates for amphibians and squamate reptiles. Nevertheless, we acknowledge that further studies will be needed to fully elucidate the effect of age at death for individual specimens.

### Getting Big Fast and Mechanisms of Cancer Defense in Birds and Mammals.

Our results show that Peto’s paradox is false—larger species have an increased prevalence of malignancy compared to smaller species. Despite being in line with molecular and physiological expectations (3), this finding appears to contradict the idea that larger body size is advantageous (21) and is at odds with Cope’s rule (22), the observed long-term trend toward increased body size through time.

Numerous studies have shown that body size evolution is a complex and highly heterogeneous process with rates of body size evolution varying greatly within different taxonomic classes and groups (16, 23–25). This means that certain species have undergone more body size evolution than would be expected, likely as a result of increased selective pressures. Across all four vertebrate classes, we find evidence for Cope’s rule (*SI Appendix*, Fig. S6), that larger species have undergone repeated instances of accelerated body size evolution (26). Body size evolution is also associated with the evolution of multiple traits, e.g., metabolic rate (27), longevity (28), etc. As a result, we might expect that high rates of body size evolution will also reflect the evolution of better anticancer mechanisms. For instance, evolution favors larger-bodied animals as is expected under Cope’s rule. Thus, we would expect that those animals that get big will have developed superior mechanisms of cancer defense. If true, we would expect to see a steeper slope between the prevalence of malignancy and body size after controlling for the differential investment in cancer defense—the heterogeneity in historic body size evolution.

### Testing Fast Rates of Body Size Evolution.

To test this hypothesis, we collected a posterior distribution of body size rate-scaled phylogenetic trees for each of the four vertebrate classes (16). Longer branches within the rate-scale phylogenetic trees represent instances in which more body size evolution has occurred than would be expected given the amount of time that has elapsed (23). We then summed the individual branch lengths from root to tip for each species to quantify the total amount of historic body size evolution that has occurred, henceforth referred to as the pathwise rate (26). Thus, pathwise rate provides an estimate of the speed of body size evolution each species has undergone throughout their evolutionary history (*Materials and Methods*).

We tested the effect of pathwise rate by fitting an MPGLMM in which neoplasia and malignancy were dependent on the number of necropsies, body size, and pathwise rate. A single slope was estimated for the number of necropsies and body size for each dependent variable for both amphibians and squamate reptiles as well as birds and mammals. Separate intercepts and slopes were estimated for pathwise rate for each class and each dependent variable.

We found that there was no significant difference between pathwise rate slopes for neoplasia in amphibians and squamate reptiles (P_x|diff_ = 0.269, *SI Appendix*, Fig. S9*A*) or birds and mammals (P _x|diff_ = 0.103, *SI Appendix*, Fig. S9*B*). Likewise, we found that there was no significant difference between pathwise rate slopes for malignancy in amphibians and squamate reptiles (P_x|diff_ = 0.355, *SI Appendix*, Fig. S9*C*) or birds and mammals (P_x|diff_ = 0.212, *SI Appendix*, Fig. S9*D*). As a result, we refitted a single pathwise rate slope for amphibians and squamate reptiles and a single slope for birds and mammals, the intercepts remained separate for each class.

We found a significant negative association between both neoplasia and pathwise rate (P_x_ = 0.027, β = −0.528, 95% CI = −1.022 to 0.017, Fig. 2*A*) and malignancy and pathwise rate (P_x_ = 0.01, β = −0.811, 95% CI = −1.463 to −0.213, Fig. 2*C*) in birds and mammals. This means that birds and mammals that underwent more total body size evolution have a decreased prevalence of neoplasia and malignancy even if their present-day body size is the same. In contrast, we found that pathwise rate was not a significant predictor of neoplasia (P_x_ = 0.051, β = 1.297, 95% CI = −0.185 to 3.007, *SI Appendix*, Fig. S10*A*) or malignancy in amphibians and squamate reptiles (P_x_ = 0.153, β = 0.880, 95% CI = 0.716 to 2.795, *SI Appendix*, Fig. S10*B*). We also tested the effect of species longevity with a single slope estimated for birds and mammals and found that species longevity was not a significant predictor of either neoplasia (P_x_ = 0.132, β = 0.168, 95% CI = −0.145 to 0.486, *SI Appendix*, Fig. S11*A*) or malignancy (P_x_ = 0.107, β = 0.127, 95% CI = −0.261 to 0.535, *SI Appendix*, Fig. S11*B*). Similarly, we also tested the effect of age at death with a single slope estimate for birds and mammals and found that age of death was not a significant predictor of either neoplasia (P_x_ = 0.232, β = 0.130, 95% CI = −0.230 to 0.479, *SI Appendix*, Fig. S12*A*) or malignancy (P_x_ = 0.323, β = 0.112, 95% CI = −0.380 to 0.597, *SI Appendix*, Fig. S12*b*).

Consistent with our previous findings (Fig. 1), we found a significant positive association between both neoplasia and body mass (P_x_ = 0, β = 0.150, 95% CI = 0.068 to 0.233, Fig. 2*B*) and malignancy and body mass (P_x_ = 0.001, β = 0.158, 95% CI = 0.065 to 0.256, Fig. 2*D*) with the inclusion of pathwise rate as an independent variable in the model. However, the gradient of the body mass slope increased by 16% for neoplasia and 27% for malignancy in birds and mammals compared to the body mass-only model (Fig. 1), supporting the hypothesis that high rates of body size evolution may reflect the evolution of more superior anticancer mechanisms. That is, after accounting for the variation in the rate of historic body size evolution, and thus potential anticancer mechanisms, there is a greater increase in neoplasia and malignancy prevalence in birds and mammals with respect to an increase in species size. Finally, we found that body mass and pathwise rate explained a significant proportion of the variation in neoplasia and malignancy (marginal R^2^ = 0.688 and conditional R^2^ = 0.827), further demonstrating the extra explanatory power of the pathwise rate covariate.

## Discussion

Peto’s paradox has remained one of the most elusive mysteries in comparative biology (6, 12, 29). Here, we combine a broad pan-species prevalence dataset (13) with a statistically robust regression framework (18) to reveal a positive association between malignancy and body size thus providing no support for Peto’s paradox: Larger species have an increased prevalence of cancer compared to smaller species. Similarly, while we found that *Elephas maximus,* the Asian elephant, has a lower than excepted prevalence of malignancy, we found no evidence to suggest that it has an exceptionally low prevalence of malignancy despite often being touted as the quintessential example of Peto’s paradox (7, 9).

**Fig. 3.**
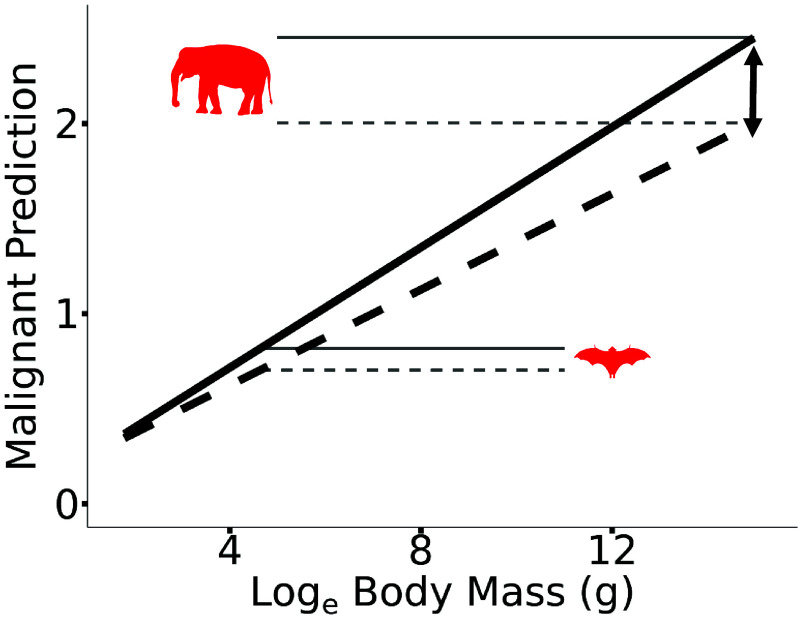
A schematic depiction of how high rates of body mass evolution change the scaling of malignancy prevalence with body mass. The black dotted line shows the mean average predicted slope from a model with body mass as the only fixed effect. The black solid line shows the mean average predicted slope from a model with body mass and pathwise rate as fixed effects. The slope of the black solid line is plotted with the pathwise rate set equal to the pathwise rate of the non-rate-scaled tree. The slope of the solid line is steeper than the slope of the dotted line, meaning that after accounting for high rates of body mass evolution, there is a greater increase in malignancy prevalence with respect to an increase in species body mass. As a result, larger species experience a greater reduction in the predicted prevalence of malignancy compared to smaller species. For instance, high rates of body size evolution are associated with a 56% reduction in the predicted prevalence of malignancy in *Elephas maximus* but only a 12% reduction in *Rousettus lanosus*, as shown by the gray dotted and solid lines.

The mix of substrates used by natural selection to sculpt and shape species body size through time has also resulted in concomitant solutions (30) that stave off the growing threat of cancer as birds and mammals become ever larger. As a result, larger species of birds and mammals have grown in size without incurring the same increase in cancer prevalence as would be expected compared to smaller species. That is, the rate of body mass evolution may be important for reduced cancer prevalence (7, 31, 32). For instance, high rates of body mass evolution were associated with a 56% reduction in the predicted prevalence of malignancy in *E. maximus,* the Asian elephant. In contrast, *Rousettus lanosus,* the long-haired fruit bat, is over 30,000 times smaller than *E. maximus,* and high rates of body mass evolution are only associated with a 12% reduction in the predicted prevalence of malignancy (Fig. 3). In practical terms, this means that *E. maximus* has the expected malignancy prevalence of an animal that is only 10% of its body size, such as *Panthera tigris,* the tiger.

The evolution of endothermy in birds and mammals is one of the most dramatic and important transitions in vertebrate evolution driving widespread expansion and ecological success (33). However, the increased metabolic cost incurred with endogenous body temperature control has also been linked to the loss of regenerative tissue capabilities (34). As a result, we speculate that this loss of this capability may also explain the lack of association between high rates of body size evolution and malignancy prevalence in amphibians and reptiles. That is, while natural selection blanketly tightened the vice of cellular growth control in birds and mammals to permit a continual increase in size, it may have simply tweaked and tuned the regulation in amphibians and reptiles to allow for the regeneration of ever larger limbs. If true, then we would hypothesize that high rates of body size evolution may in fact be positively associated with malignancy prevalence in a subset of ectothermic species with enhanced regenerative tissue capabilities e.g., urodele amphibians. That is, the cellular mechanisms underlying regenerative capability could be potentially high jacked driving malignant growth.

**Fig. 4.**
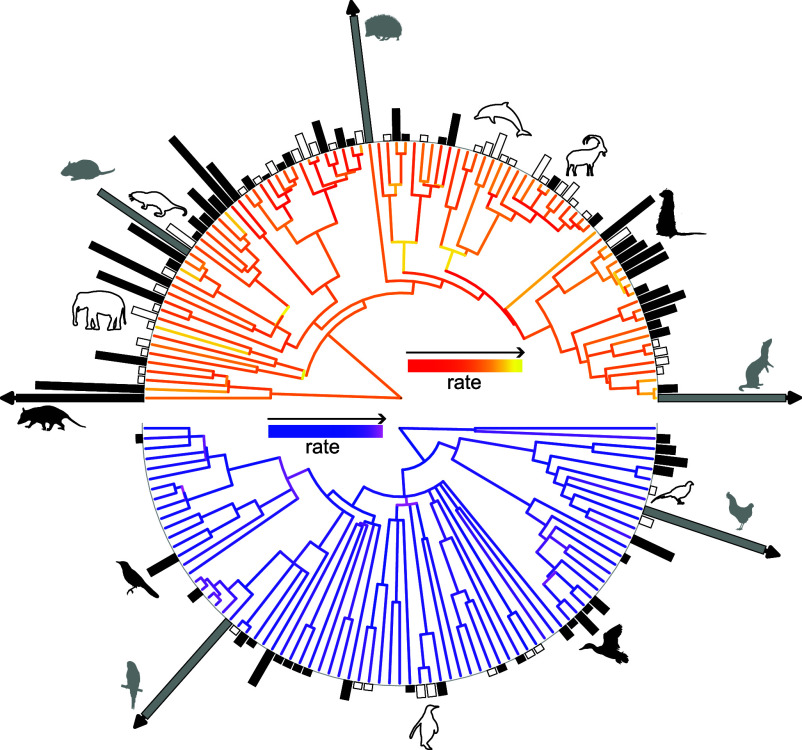
Predicted malignancy prevalence across birds and mammals. The branches of the phylogeny are colored with respect to the rate of body mass evolution, lighter shades correspond to faster rates. The bars at the tips of the phylogeny correspond to the difference between the predicted and observed prevalence of malignancy in birds and mammals with respect to body mass and pathwise rate. The solid black bars and white bars indicate species with a higher and lower-level prevalence of malignancy than would be expected. The gray bars indicate outlier species that were removed during the model fitting (*Materials and Methods*). The black arrows indicate species in which the prevalence difference is greater than 20.

On average, larger species are expected to have an increased prevalence of malignancy compared to smaller species (Figs. 1 and 2). However, species-to-species variation means that some species will still get more or less malignancy than expected. To identify which species were the winners and losers of the antimalignancy arms race we compared the observed and predicted prevalence of malignancy in birds and mammals with respect to body size and pathwise rate (*Materials and Methods*). We found that a total of 116 out of 169 species had a predicted prevalence that differed from their observed prevalence and that more species had a higher-than-expected prevalence in both birds (28/38) and mammals (42/78) (Fig. 4). We found that *Melopsittacus undulatus*, the budgerigar, a species known to have a high prevalence of neoplasia (35), had the highest prevalence of malignancy and the greatest underestimate of malignancy prevalence given its body size and pathwise rate. In contrast, *Heterocephalus glaber,* the naked mole rate, had the greatest overestimate of malignancy, thus highlighting its superior anticancer defense mechanisms (36). These results highlight subsets of species that, despite their body size and historic rate of body size evolution, have an exceptionally high or low prevalence of malignancy and thus serve as a target group for future mechanistic studies of malignancy defense.

## Materials and Methods

### Data and Code Availability.

Neoplasia and malignancy prevalence data for each species are available from Compton et al. (13). As outlined by Compton et al, all necropsies were performed by specialist veterinary pathologists and neoplasms were identified by board-certified pathologists. A single phylogenetic tree from Compton et al. (13), originally collected from timetree.org, was used across all four vertebrate classes. Body size data (body mass for birds and mammals and SVL for amphibians and squamate reptiles) are available from Cooney and Thomas (16), as are the posterior distributions of the body size rate-scaled phylogenetic trees for each of the four vertebrate classes. The total amount of body size evolution, henceforth referred to as the pathwise rate, was calculated for each species by summing the individual branch lengths from root to tip for each rate-scaled tree in the posterior distribution using the distRoot function in the adephylo R package (37). The median value for each species was then calculated across the 10,000-tree posterior distribution. The final dataset with paired prevalence and body size data contained 263 species: 31 amphibians, 79 birds, 90 mammals, and 63 reptiles. The necessary code to calculate the pathwise rate for each species and fit the MPGLMMs outlined below is available at https://github.com/george-butler/terrestrial_vertebrate_cancer. The software BayesTraitsV4 (38) used to evaluate the association between pathwise rate and body size is available at https://www.evolution.reading.ac.uk/BayesTraitsV4.1.1/BayesTraitsV4.1.1.html.

### MPGLMMs.

Neoplasia encompasses benign and malignant tumors as well as tumors that are not yet malignant but have the potential for malignant transformation, known as premalignancies. The transient and dynamic nature of premalignancies poses an analytical challenge when testing Peto’s paradox due to the presence of two transition probabilities (e.g., normal state → premalignant state → malignant state) (39). As a result, attempts have been made to account for the prevalence of premalignancies by quantifying the rate of malignancy as the number of animals with malignancy relative to the number of animals with neoplasia, rather than the number of animals sampled (11, 39). However, certain species have a higher prevalence of certain cancer types (e.g., carcinoma vs sarcoma)(13). Thus, the rate at which premalignant tumors transform to malignant tumors is expected to differ. As a result, quantifying the rate of malignancy as a proportion relative to the number of animals with neoplasia will result in an underestimation of the rate of premalignant to malignant transformations. In contrast, a multivariate regression framework allows the rate of malignancy to be quantified with respect to the number of animals with neoplasia and the number of animals sampled while ensuring enough flexibility to account for heterogeneity in the rate of premalignant to malignant transformations. This structure allows us to explicitly incorporate the number of necropsies as an explanatory variable in our models (see below).

#### Model structures.

We used MPGLMMs throughout that were fitted in a Bayesian Markov Chain Monte Carlo (MCMC) framework using the MCMCglmm (18) R package. Specifically, neoplasia and malignancy prevalence were estimated jointly in each stage of analysis with separate models fitted for amphibians and reptiles (model 1) and for birds and mammals (model 2). Separate intercepts were estimated for each class in each stage of analysis. Phylogeny was included as a random effect in every model to account for the shared ancestry.

In the first stage of analysis, as shown in Fig. 1, the log_e_ total number of necropsies and log_e_ body size were included as fixed effects, outlined in Model 1. A single slope was estimated for the log_e_ total number of necropsies for each dependent variable in each model. A complex model was first fitted in which separate body size slopes were estimated for each class and each dependent variable. However, no significant difference was found in the body size slopes in either model for either dependent variable (*SI Appendix*, Fig. S5). As a result, a reduced model was fitted with a single body size slope estimated for each model and each dependent variable.

*Model 1:*

Neoplasia and Malignancy = Log_e_ (Number of Records) + Log_e_ (Body Mass) * Class + Phylogeny.

In the second stage of analysis, as shown in Fig. 2, the log_e_ total number of necropsies, log_e_ body size, and the log_e_ pathwise rate (see above) were included as fixed effects, outlined in Model 2. A single slope was estimated for the log_e_ total number of necropsies and log_e_ body size in each model for each dependent variable. A complex model was first fitted in which separate pathwise rate slopes were estimated for each class and each dependent variable. However, no significant difference was found in the pathwise rate slopes in either model for each dependent variable (*SI Appendix*, Fig. S9). As a result, a reduced model was fitted with a single pathwise rate slope estimated for each model and for each dependent variable.

*Model 2:*

Neoplasia and Malignancy = Log_e_ (Number of Records) + [Log_e_ (Body Mass) + Log_e_ (Pathwise Rate)] * Class + Phylogeny.

#### MCMC conditions.

All MCMC chains were run for 10^6^ iterations with sampling at every 1,000th iteration after visual convergence was achieved. We fitted all MPGLMMs with a Poisson link to account for the error structure in the neoplasia and malignancy count data. MCMCglmm automatically accounts for overdispersion in count data. We used default priors ($\mu ={\vec{0}}_{n}$ and V = **I**_n_ × 10^8^, where ${\vec{0}}_{n}$ is the zero vector and **I**_n_ is the identity matrix in which n is equal to the number of fixed effects in the model) for the fixed effects and multivariate parameter-expanded priors (V = **I**_2_ * 2, ν = 2, $\alpha \mu ={\vec{0}}_{2}$, and αV = **I**_2_ * 25^2^, where ${\vec{0}}_{2}$ is the two-dimensional zero vector and is the **I**_2_ two-dimensional identity matrix) for the phylogenetic random effects (40). After convergence was achieved, the posterior distribution for each independent variable as well as the random effect was visually assessed to ensure model assumptions were met.

#### Assessing significance and quantifying the variation explained by the model (R**^2^**).

Regression parameter significance was assessed by the proportion of the posterior distribution that crosses zero (P_x_), where P_x_ < 0.05 is considered to be significantly different from 0. To evaluate whether the magnitude of a fixed effect was significantly different between classes (Figs. 1 and 2) we compared the estimated slopes at each iteration and assessed the proportion of the posterior distribution of pairwise differences that cross zero (P_x|diff_). If P_x|diff_ < 0.05 two parameters are considered to be significantly different and thus distinct.

The variation explained by models 1 and 2 (Figs. 1 and 2 respectively) was measured by calculating a pseudo-R^2^ for each model. Specifically, the marginal and conditional R^2^ values were calculated using the method outlined by Nakagawa et al. (41).

#### Visualizing fitted models.

The individual data points in each model are not independent. They share varying amounts of ancestry as implied by the phylogeny. However, it is not possible to visualize the individual data points after accounting for the level of relatedness. Thus, to limit potential confusion, the individual data points are not included with the fitted models in Figs. 1 and 2 or *SI Appendix*, Figs. S1 and S12. As a result, the y-axis is labeled as “Neoplasia prediction” or “Malignancy prediction” to indicate that it is an estimate from the model rather than a sampled value. Put simply, the y-axis can be thought of as the number of neoplasia or malignancy expected for a certain value on the x-axis (e.g., for a given body size or pathwise rate) given the estimated model. Finally, the fitted models from Figs. 1 and 2 are shown with the data points included in *SI Appendix*, Figs. S13 and S14. Owing to the number of species with zero prevalence of neoplasia or malignancy the data and fitted models are shown on a non-log-transformed Y-axis, hence the nonlinearity. Crucially, the parameters from the fitted models in *SI Appendix*, Figs. S13 and S14 are estimated from regression models (models 1 and 2 outlined above) that included multiple fixed effects and phylogeny as a random effect. As a result, caution should be taken before drawing conclusions based upon visual inspection.

### Detecting Potential Outliers.

To ensure that our results were not driven by a small number of potential outlier species, we removed species with a studentized residual greater than three (42). The studentized residual for species *i* was calculated by refitting the model with the *i*th species removed. The *i*th species residual was then quantified as the difference between the observed and predicted prevalence where the prediction is conditional on the estimates of the fixed effects only. The studentized residual for species *i* was then calculated by dividing the *i*th species residual by its estimated standard deviation (42).

We calculated the studentized residual for each individual species in both the first and second stage of analysis, shown in Figs. 1 and 2. A species was classified as a potential outlier, and removed from the data, if the studentized residual was greater than three for either neoplasia or malignancy. Six species of amphibians and reptiles were identified as potential outliers: *Heloderma suspectum*, *Lampropeltis getula*, *Lampropeltis triangulum*, *Leptodactylus fallax*, *Pantherophis guttatus*, and *Peltophryne lemur*. Five species of birds or mammals were identified as potential outlier: *Atelerix albiventris*, *Gallus gallus*, *Melopsittacus undulatus*, *Mustela putorius*, and *Rattus norvegicus*. The same 11 species were identified as potential outliers in both the first and second stage of analysis (Figs. 1 and 2).

### Longevity and Age at Death Analysis.

To test the effect of species longevity we collected longevity information from The Animal Ageing and Longevity Database (AnAge) (19). A total of 177 species within our 252 outlier removed species dataset had matched longevity information: 12 amphibians, 45 birds, 83 mammals, and 37 squamate reptiles.

First, we fitted separate MPGLMMs for amphibians and squamate reptiles as well as birds and mammals with neoplasia and malignancy prevalence dependent on the log_e_ total number of necropsies, log_e_ body size, and log_e_ longevity. In each model, a single slope was estimated for each of the fixed effects and phylogeny was included as a random effect.

We found that species longevity was not a significant predictor of neoplasia in amphibians and squamate reptiles (P_x_ = 0.1, *SI Appendix*, Fig. S4*A*) or birds and mammals (P_x_ = 0.245, *SI Appendix*, Fig. S4*B*). Likewise, species longevity was not a significant predictor of malignancy in amphibians and squamate reptiles (P_x_ = 0.118, *SI Appendix*, Fig. S4*C*) or birds and mammals (P_x_ = 0.21, *SI Appendix*, Fig. S4*D*). Next, we fitted a second MPGLMM for birds and mammals with log_e_ pathwise-rate included as an additional independent variable. Consistent with our previous findings, we found that species longevity was not a significant predictor of either neoplasia or malignancy (P_x_ = 0.132 & 0.107 respectively, *SI Appendix*, Fig. S11).

To evaluate the effect of age at death we used the age of death at the time of necropsy from Compton et al. (13). Age at death information was not publicly available for all species in which a necropsy had been conducted. Thus, for a species to be included in our dataset, we required that age at death needed to have been recorded for at least 20 specimens. The median age was then calculated for each species. A total of 73 species within our 252 outlier removed species dataset had matched age at death information: 1 amphibian, 12 birds, 49 mammals, and 11 squamate reptiles. The limited number of amphibians and squamate reptiles meant that further analysis was not feasible in these classes.

In birds and mammals, we fitted an MPGLMM in which neoplasia and malignancy prevalence were dependent on the log_e_ total number of necropsies, log_e_ body size, and the log_e_ age at death. A single slope was estimated for each fixed effect with phylogeny included as a random effect. We found that species age at death was not a significant predictor of either neoplasia or malignancy (P_x_ = 0.221 & 0.302 respectively). In addition, we also fitted a second MPGLMM within log_e_ pathwise rate included as an additional independent variable. Again, we found that species age at death was not a significant predictor of either neoplasia or malignancy (P_x_ = 0.232 & 0.323 respectively).

### Detecting an Association Between Pathwise Rate and Body Size.

To test for an association between pathwise rate and body size we fitted a univariate phylogenetic generalized least squares model in a MCMC framework across the 263 species dataset using BayesTraitsV4 (38). Specifically, we regressed log_e_ pathwise rate against the log_e_ SVL in amphibians and reptiles and the log_e_ body mass in birds and mammals. We used default priors and the MCMC chains were run for 20^6^ iterations with sampling every 10^4^ iterations after convergence. A separate intercept was estimated for each class and phylogenetic signal (λ) was estimated for each model.

We found a positive association between pathwise rate and SVL in amphibians and reptiles (*SI Appendix*, Fig. S6*A*). Similarly, we found a significant positive association between pathwise rate and body mass in birds and mammals (*SI Appendix*, Fig. S6*B*). Across all four vertebrate classes, larger species have an increased pathwise rate, thus demonstrating that on average larger species have on average undergone more total historic body size evolution compared to smaller species.

### Identifying Birds and Mammals with Exceptionally High or Low Prevalence of Malignancy.

We identified birds and mammals with an “exceptional” prevalence of malignancy, either high or low, by comparing the species observed prevalence of malignancy to their expected level given their body size and pathwise rate. Specifically, we fitted an MPGLMM with neoplasia and malignancy dependent on the log_e_ number of necropsies, log_e_ body mass, and log_e_ pathwise rate. A single slope was estimated for each of the fixed effects for each dependent variable and separate intercepts were estimated for each class. Phylogeny was included as a random effect to account for the shared ancestry. The model was then fitted with the 5 potential outlier species of birds and mammals (listed above) removed. The number of MCMC iterations, sampling frequency, and prior specification were kept the same.

The fitted model was then used to predict the prevalence of malignancy for each of the 164 species of birds and mammals that were present during the model estimation process as well as the 5 species removed as potential outliers. The predictions were made conditional on the estimates of the fixed and random effects resulting in an unlogged predictive posterior distribution. Finally, the median value was calculated for each species and compared to the observed prevalence to identify species with a higher or lower prevalence of malignancy than expected given their body mass and pathwise rate.

## Supplementary Material

Appendix 01 (PDF)

## Data Availability

Path-wise rate for each species and fit the Multivariate Phylogenetic Generalized Linear Mixed Models (MPGLMMs); software BayesTraitsV4 (38) used to evaluate the association between path-wise rate and body size data have been deposited in github; (https://github.com/george-butler/terrestrial_vertebrate_cancer; https://www.evolution.reading.ac.uk/BayesTraitsV4.1.1/BayesTraitsV4.1.1.html). Previously published data were used for this work (13, 16).
