# Supplementary material for: No evidence for Peto’s paradox in terrestrial vertebrates
Source: Proc Natl Acad Sci U S A. 2025 Feb 24;122(9):e2422861122. doi: 10.1073/pnas.2422861122 (PMC11892590; doi:10.1073/pnas.2422861122)
Supplement: Supplementary file 1 — Appendix 01 (PDF) [file pnas.2422861122.sapp.pdf]

Supporting Information for
No evidence for Peto's Paradox in terrestrial vertebrates

Authors: George Butler, Joanna Baker, Sarah R. Amend, Kenneth J. Pienta, Chris Venditti

Corresponding authors: George Butler & Chris Venditti
Email: george-butler@ucl.ac.uk & c.d.venditti@reading.ac.uk

This PDF file includes:

Figures S1 to S14

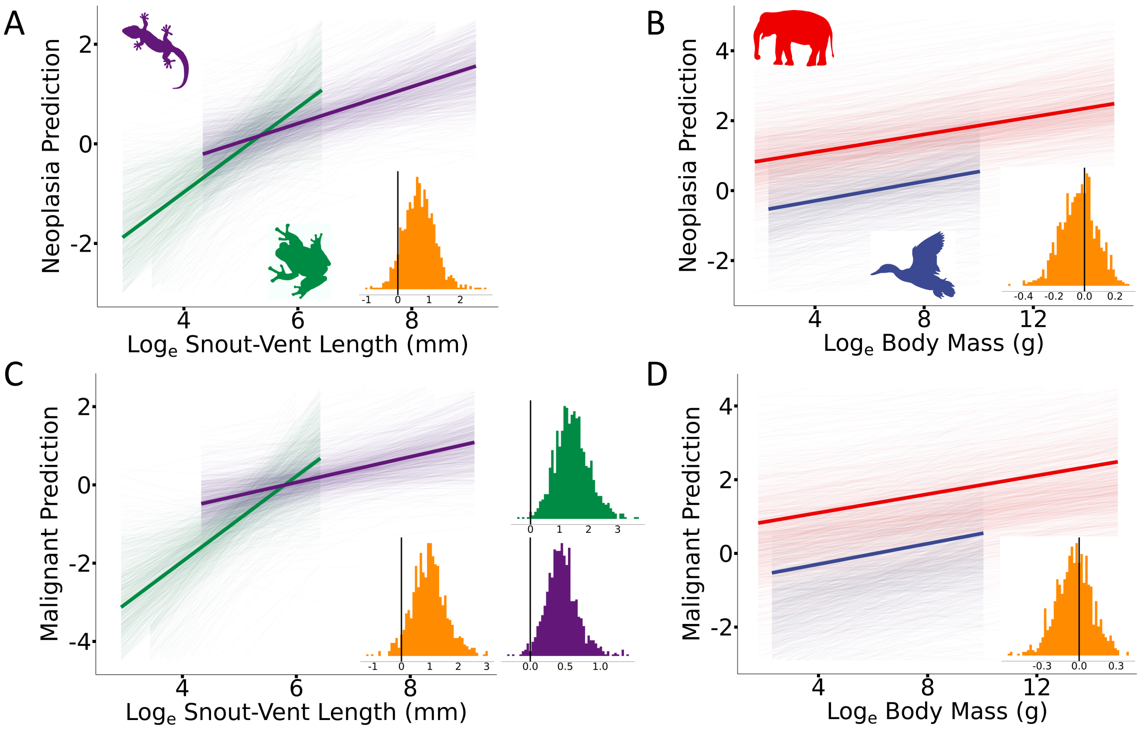

Fig. S1. Testing for a class specific association between neoplasia or malignancy and body size with outlier species included. In all cases, the posterior predicted slopes are plotted, and the mean average predicted slopes are highlighted. Orange Insets show the posterior distribution of differences between the estimated slopes, two slopes are significantly different if less than 5% of the pairwise posterior distribution crosses 0. The green and purple Insets show the posterior distribution of the estimated slopes for amphibians and reptiles respectively, a slope is significant if less than 5% of the posterior distribution crosses 0. The black vertical line indicates 0 on the x-axis. (**A**) There is no significant difference in the neoplasia slopes between amphibians (green) and squamate reptiles (purple) ($P_{x|diff}$ = 0.073) or (**B**) birds (blue) and mammals (red) ($P_{x|diff}$ = 0.380) . (**C**) The malignancy slope is significantly steeper for amphibians compared to squamate reptiles ($P_{x|diff}$ = 0.046) and both amphibians and squamate reptiles have a significant positive association between malignancy and SVL (P_x = 0.003 & 0.018 respectively). (**D**) There is no significant difference in the malignancy slopes between birds and mammals ($P_{x|diff}$ = 0.391).

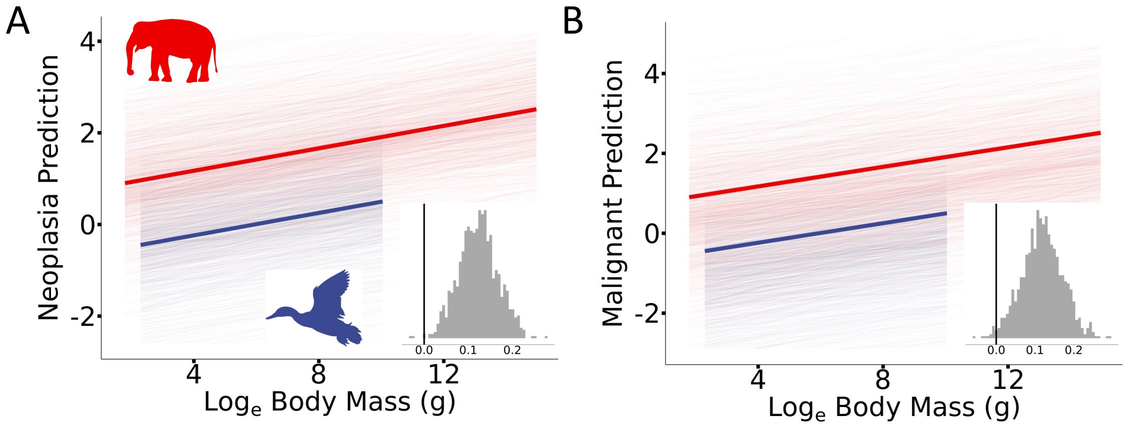

Fig. S2. A positive association between neoplasia or malignancy and body mass in birds and mammals with outlier species included. In both cases, the posterior predicted slopes are plotted, and the mean average predicted slopes are highlighted. Insets show the posterior distribution of the estimated slopes, a slope is significant if less than 5% of the posterior distribution crosses 0. The black vertical line indicates 0 on the x-axis. (**A**) Neoplasia prevalence is positively associated with body mass in birds (blue) and mammals (red) (P_x = 0.003). (**B**) Malignancy prevalence is positively associated with body mass in birds and mammals (P_x = 0.012).

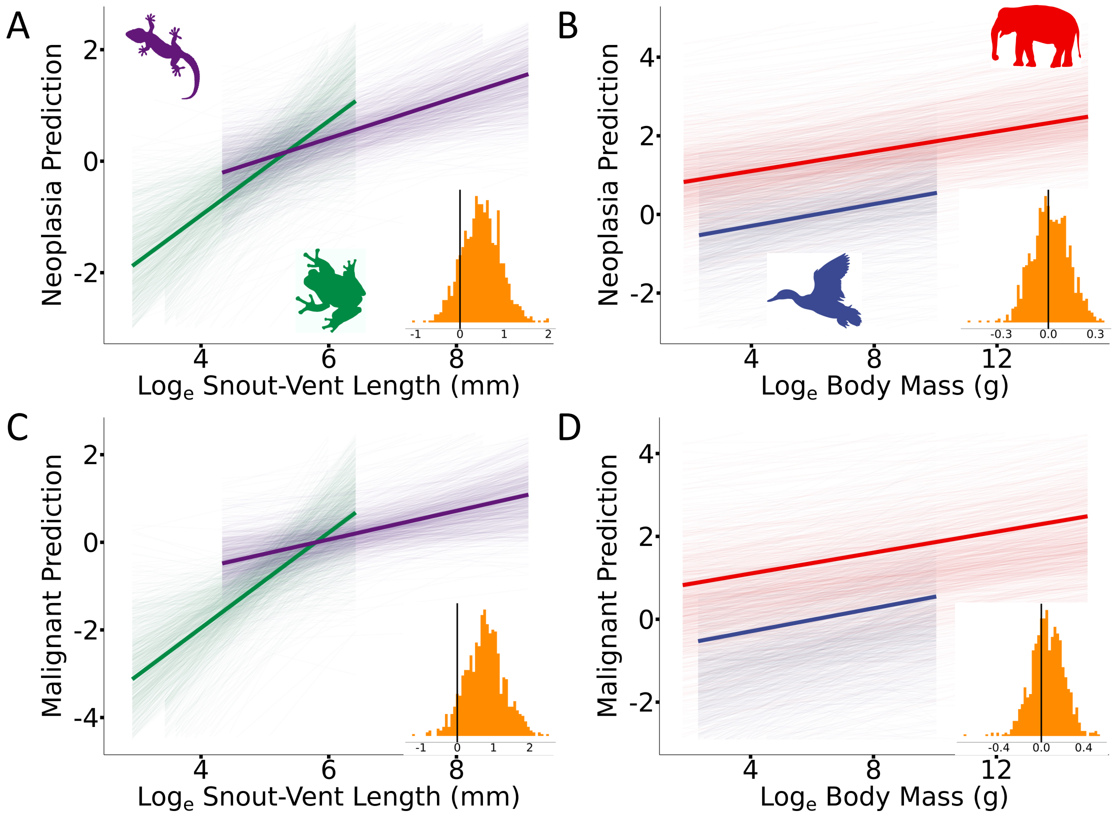

Fig. S3. Testing for a class specific association between neoplasia or malignancy and body size. In all cases, the posterior predicted slopes are plotted, and the mean average predicted slopes are highlighted. Insets show the posterior distribution of differences between the estimated slopes, two slopes are significantly different if less than 5% of the pairwise posterior distribution crosses 0. The black vertical line indicates 0 on the x-axis. There is no significant difference in the neoplasia slopes between (**A**) amphibians (green) and squamate reptiles (purple) ($P_{x|diff} = 0.138$) or (**B**) birds (blue) and mammals (red) ($P_{x|diff} = 0.456$). There is no significant difference in the malignancy slopes between (**C**) amphibians and squamate reptiles ($P_{x|diff} = 0.076$) or (**D**) birds and mammals ($P_{x|diff} = 0.344$).

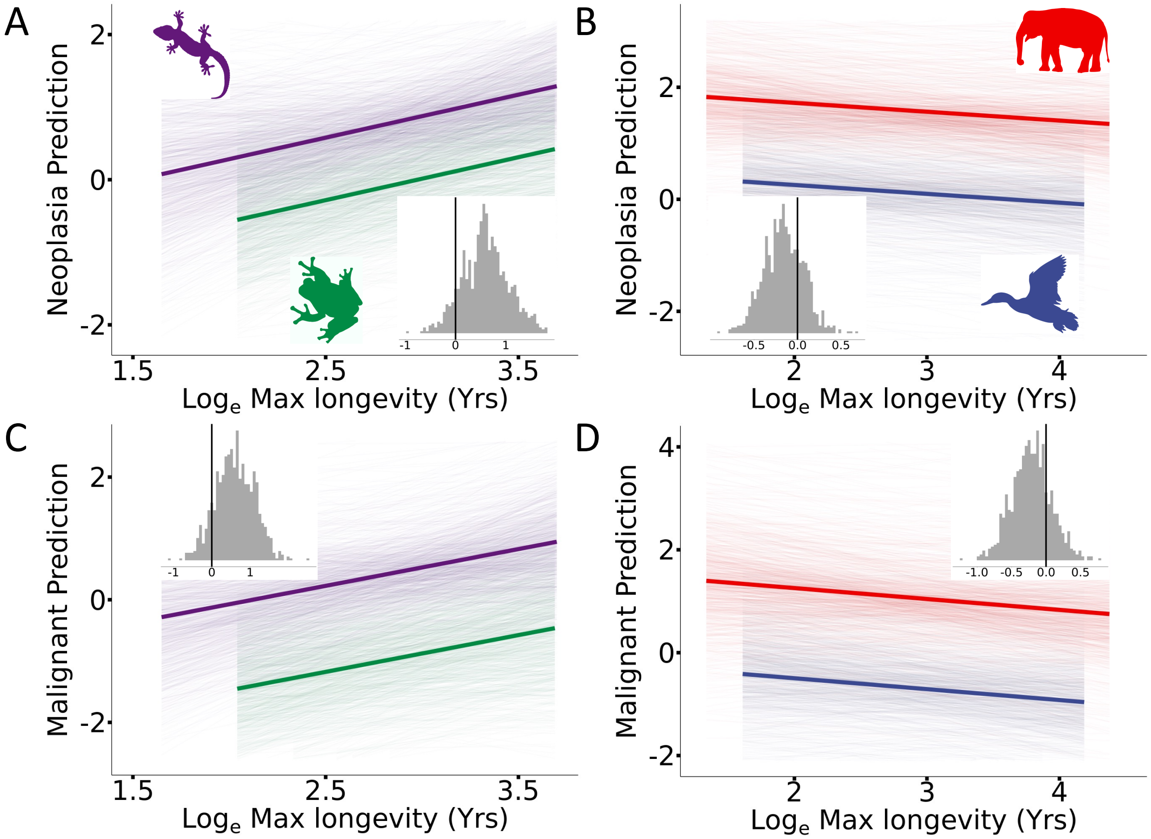

Fig. S4. No association between neoplasia or malignancy and longevity in a model with body size. In all cases, the posterior predicted slopes are plotted, and the mean average predicted slopes are highlighted. Insets show the posterior distribution of the estimated slopes, a slope is significant if less than 5% of the posterior distribution crosses 0. The black vertical line indicates 0 on the x-axis. The dataset is reduced due to limited longevity data: 12 amphibians, 45 birds, 83 mammals, and 37 squamate reptiles. Neoplasia prevalence is not significantly associated with longevity (**A**) in amphibians (green) and squamate reptiles (purple) (P_x = 0.1) or (**B**) birds (blue) and mammals (red) (P_x = 0.245). Malignancy prevalence is not significantly associated with longevity (**C**) in amphibians and squamate reptiles (P_x = 0.118) or (**D**) birds and mammals (P_x = 0.21).

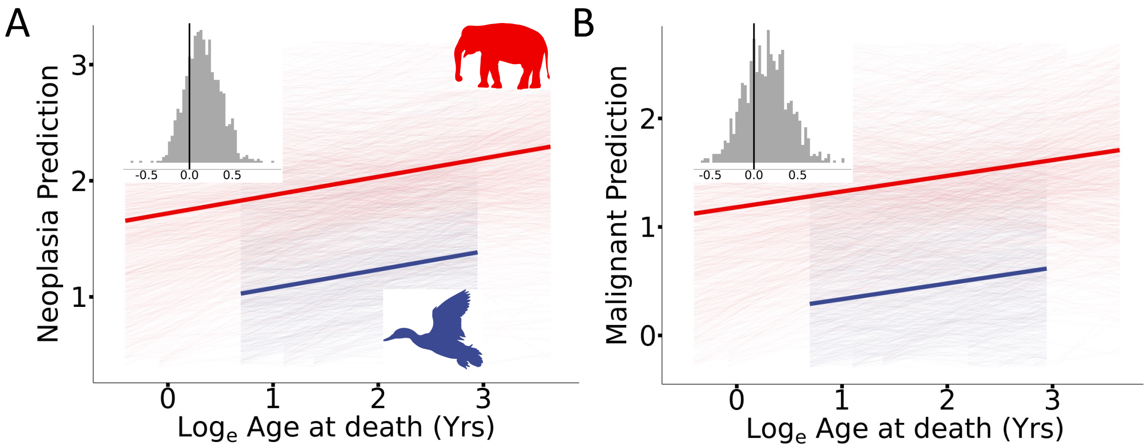

Fig. S5. No association between neoplasia or malignancy and age at death in a model with body mass. In all cases, the posterior predicted slopes are plotted, and the mean average predicted slopes are highlighted. Insets show the posterior distribution of the estimated slopes, a slope is significant if less than 5% of the posterior distribution crosses 0. The black vertical line indicates 0 on the x-axis. The dataset is reduced due to limited age at death data: 12 birds and 49 mammals. (**A**) Neoplasia prevalence is not significantly associated with longevity in birds (blue) and mammals (red) (P_x = 0.221). (**B**) Malignancy prevalence is not significantly associated with longevity in birds and mammals (P_x = 0.302).

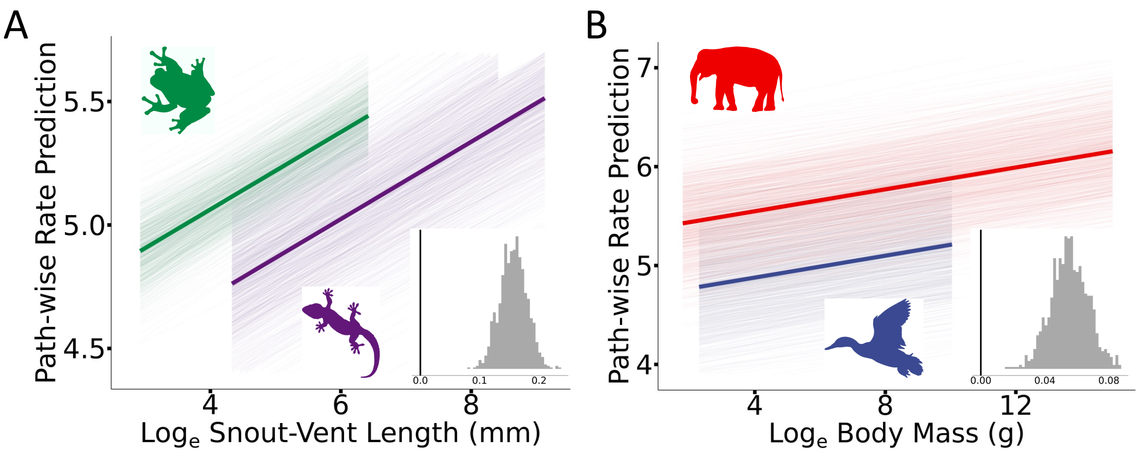

Fig. S6. A positive association between path-wise rate and body size with outliers included. In all cases, the posterior predicted slopes are plotted, and the mean average predicted slopes are highlighted. Insets show the posterior distribution of the estimated slopes, a slope is significant if less than 5% of the posterior distribution crosses 0. The black vertical line indicates 0 on the x-axis. (**A**) Path-wise rate is significantly associated with snout-vent length in amphibians (green) and squamate reptiles (purple) (P_x= 0). (**B**) Path-wise rate is significantly associated with body mass in birds (blue) and mammals (red) (P_x= 0).

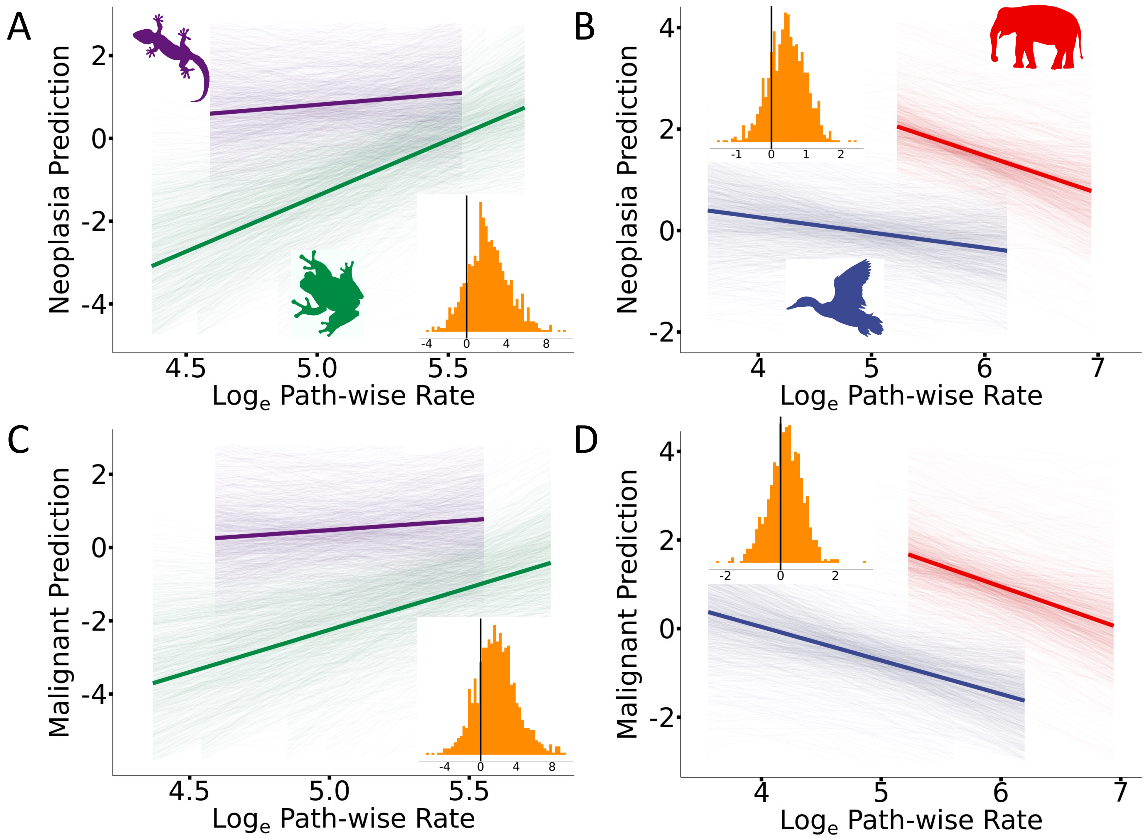

Fig. S7. Testing for a class specific association between neoplasia or malignancy and path-wise rate in a model with body mass and outlier species included. In all cases, the posterior predicted slopes are plotted, and the mean average predicted slopes are highlighted. Insets show the posterior distribution of differences between the estimated slopes, two slopes are significantly different if less than 5% of the pairwise posterior distribution crosses 0. The black vertical line indicates 0 on the x-axis. There is no significant difference in the neoplasia slopes between (**A**) amphibians (green) and squamate reptiles (purple) ($P_{x|diff} = 0.145$) or (**B**) birds (blue) and mammals (red) ($P_{x|diff} = 0.194$). There is no significant difference in the malignancy slopes between (**C**) amphibians and squamate reptiles ($P_{x|diff} = 0.205$) or (**D**) birds and mammals ($P_{x|diff} = 0.367$).

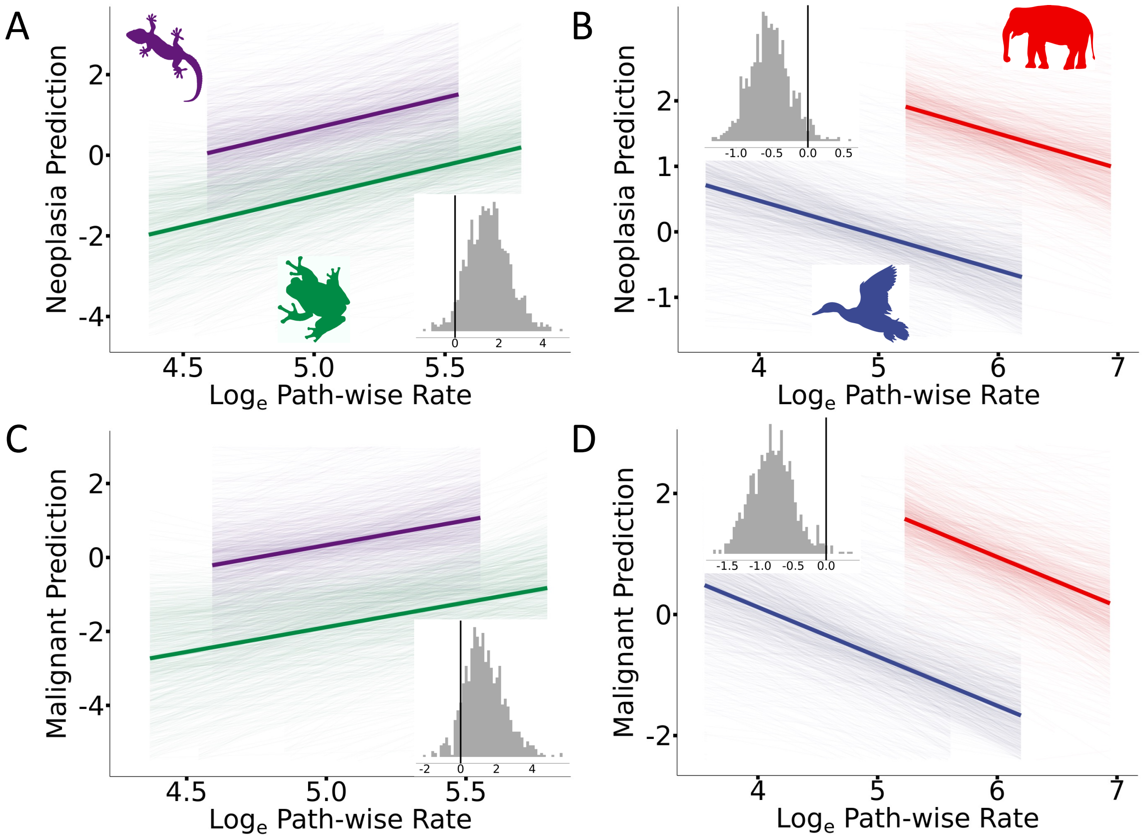

Fig. S8. Neoplasia is differentially associated with path-wise rate in a model with body mass and outlier species included. In all cases, the posterior predicted slopes are plotted, and the mean average predicted slopes are highlighted. Insets show the posterior distribution of the estimated slopes, a slope is significant if less than 5% of the posterior distribution crosses 0. The black vertical line indicates 0 on the x-axis. Separate body size slopes are estimated for amphibians (green) and squamate reptiles (purple) but only a single slope is estimated for birds (blue) and mammals (red). Neoplasia prevalence is (**A**) positively associated with path-wise rate in amphibians and squamate reptiles (P_x = 0.042) but is (**B**) negatively associated with path-wise rate in birds and mammals (P_x = 0.037). Malignancy prevalence is (**C**) not significantly associated with path-wise rate in amphibians and squamate reptile (P_x = 0.092) but is (**D**) negatively associated with path-wise rate in birds and mammals (P_x = 0.01).

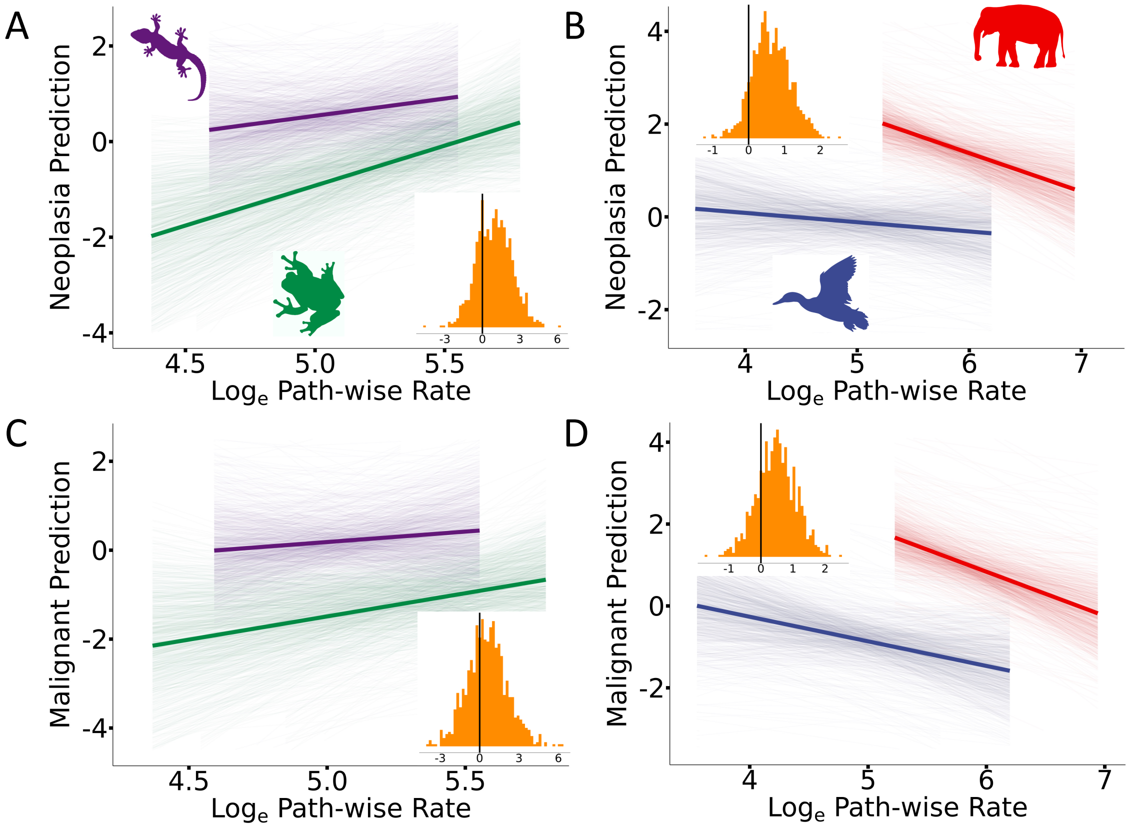

Fig. S9. Testing for a class specific association between neoplasia or malignancy and path-wise rate in a model with body mass. In all cases, the posterior predicted slopes are plotted, and the mean average predicted slopes are highlighted. Insets show the posterior distribution of differences between the estimated slopes, two slopes are significantly different if less than 5% of the pairwise posterior distribution crosses 0. The black vertical line indicates 0 on the x-axis. There is no significant difference in the neoplasia slopes between (**A**) amphibians (green) and squamate reptiles (purple) ($P_{x|diff}$ = 0.269) or (**B**) birds (blue) and mammals (red) ($P_{x|diff}$ = 0.103). There is no significant difference in the malignancy slopes between (**C**) amphibians and squamate reptiles ($P_{x|diff}$ = 0.355) or (**D**) birds and mammals ($P_{x|diff}$ = 0.212).

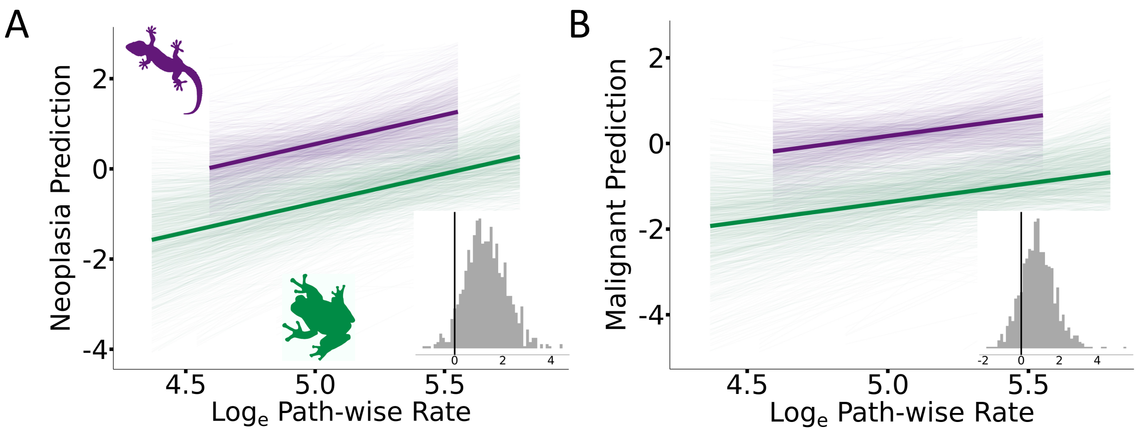

Fig. S10. No association between neoplasia or malignancy and path-wise rate in amphibians and squamate reptiles in a model with body mass. In both cases, the posterior predicted slopes are plotted, and the mean average predicted slopes are highlighted. Insets show the posterior distribution of the estimated slopes, a slope is significant if less than 5% of the posterior distribution crosses 0. The black vertical line indicates 0 on the x-axis. (**A**) Neoplasia prevalence is not significantly associated with path-wise rate in amphibians (green) or squamate reptiles (purple) (P_x = 0.051). (**B**) Malignancy prevalence is not significantly associated with path-wise rate in amphibians or squamate reptiles (P_x = 0.153).

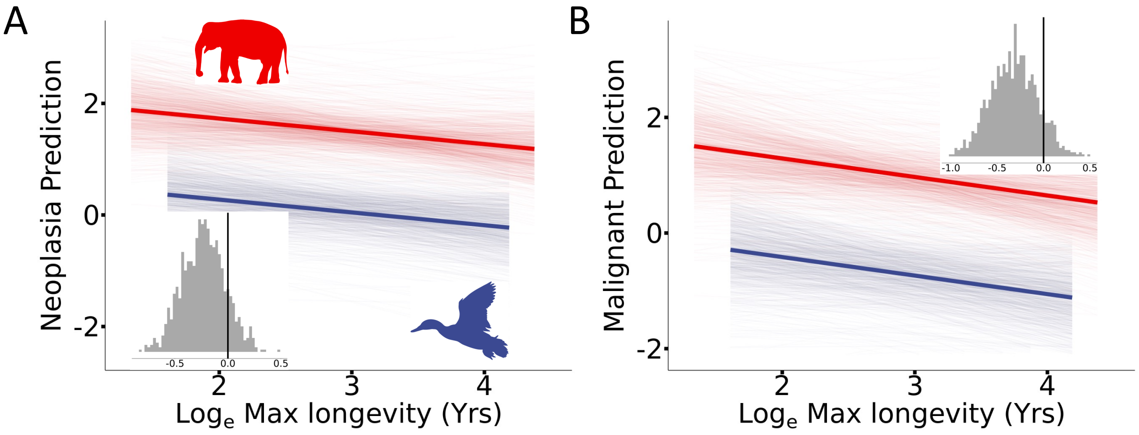

Fig. S11. No association between neoplasia or malignancy and longevity in a model with body mass and path-wise rate. In both cases, the posterior predicted slopes are plotted, and the mean average predicted slopes are highlighted. Insets show the posterior distribution of the estimated slopes, a slope is significant if less than 5% of the posterior distribution crosses 0. The black vertical line indicates 0 on the x-axis. The dataset is reduced due to limited longevity data: 45 birds and 83 mammals. (**A**) Neoplasia prevalence is not significantly associated with longevity in birds (blue) and mammals (red) (P_x = 0.132). (**B**) Malignancy prevalence is not significantly associated with longevity in birds and mammals (P_x = 0.107).

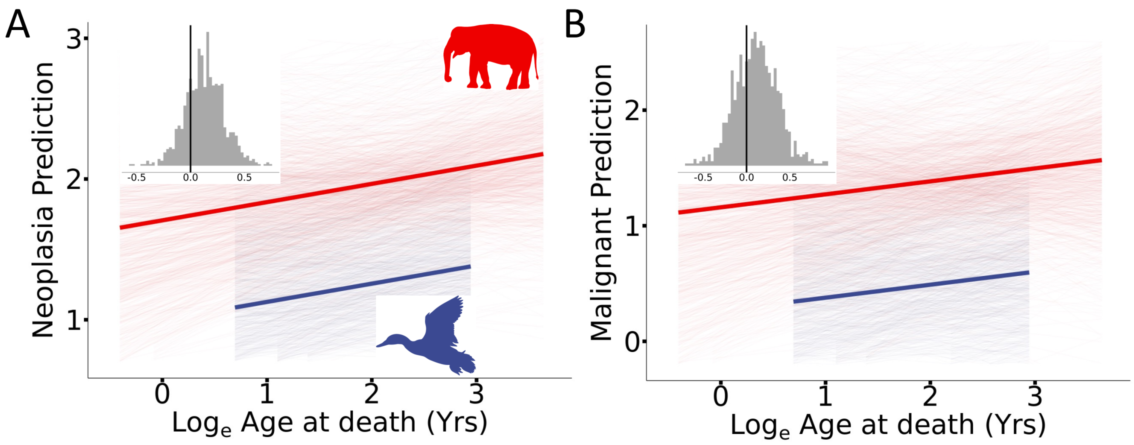

Fig. S12. No association between neoplasia or malignancy and age at death in a model with body mass and path-wise rate. In all cases, the posterior predicted slopes are plotted, and the mean average predicted slopes are highlighted. Insets show the posterior distribution of the estimated slopes, a slope is significant if less than 5% of the posterior distribution crosses 0. The black vertical line indicates 0 on the x-axis. The dataset is reduced due to limited age at death data: 12 birds and 49 mammals. (**A**) Neoplasia prevalence is not significantly associated with longevity in birds (blue) and mammals (red) (P_x = 0.232). (**B**) Malignancy prevalence is not significantly associated with longevity in birds and mammals (P_x = 0.323).

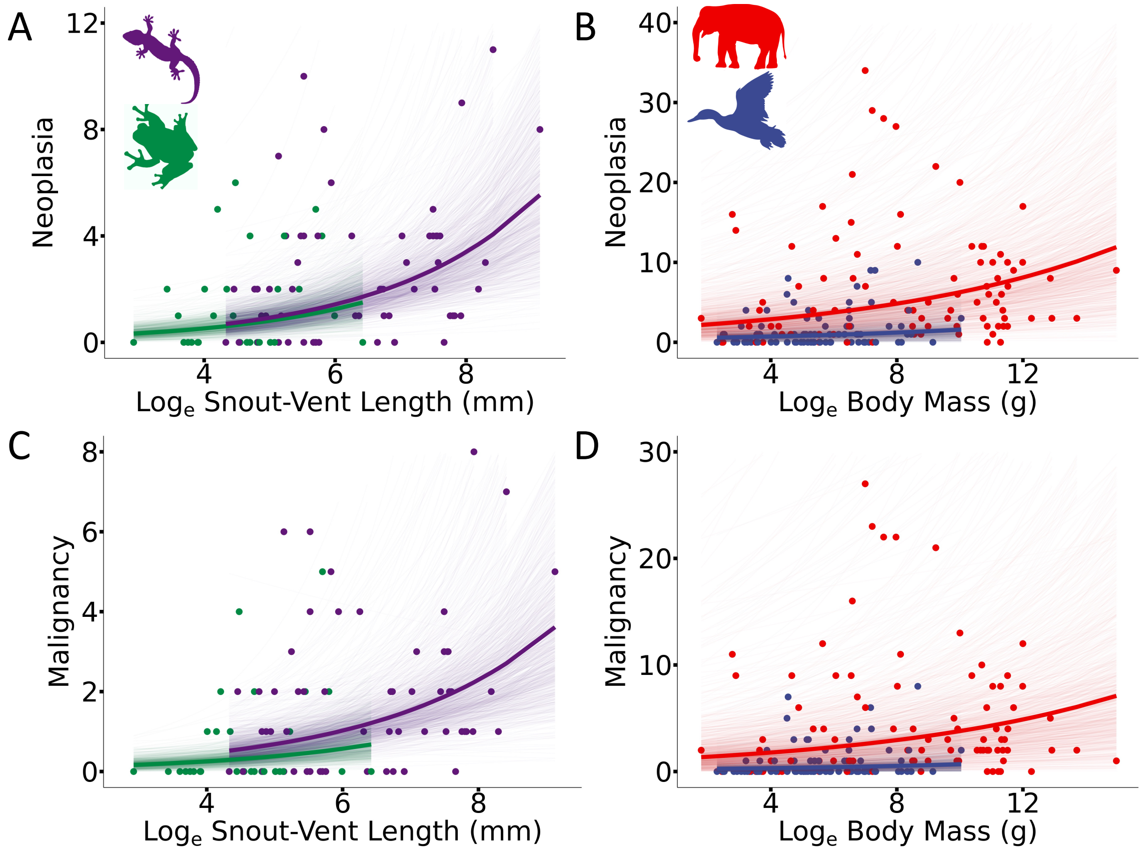

Fig. S13. A positive association between neoplasia or malignancy and body size in terrestrial vertebrates with data points included. In all cases, the posterior predicted slopes are plotted, and the mean average predicted slopes are highlighted. Neoplasia prevalence is positively associated with (**A**) snout-vent length in amphibians (green) and reptiles (purple) (P_x = 0.002) and (**B**) body mass in birds (blue) and mammals (red) (P_x = 0.001). Malignancy prevalence is positively associated with (**C**) snout-vent length in amphibians and reptiles (P_x = 0.003) and (**D**) body mass in birds and mammals (P_x = 0.001). Due to the number of species with zero prevalence of Neoplasia or Malignancy the data and fitted model are shown on a non-log transformed Y-axis, hence the non-linearity. The parameters from the fitted model are estimated for regression models that included multiple fixed effects and phylogeny as a random effect. As a result, caution should be taken before drawing conclusion based upon visual inspection. The same results are presented without data points on the log-link scale in Figure 1.

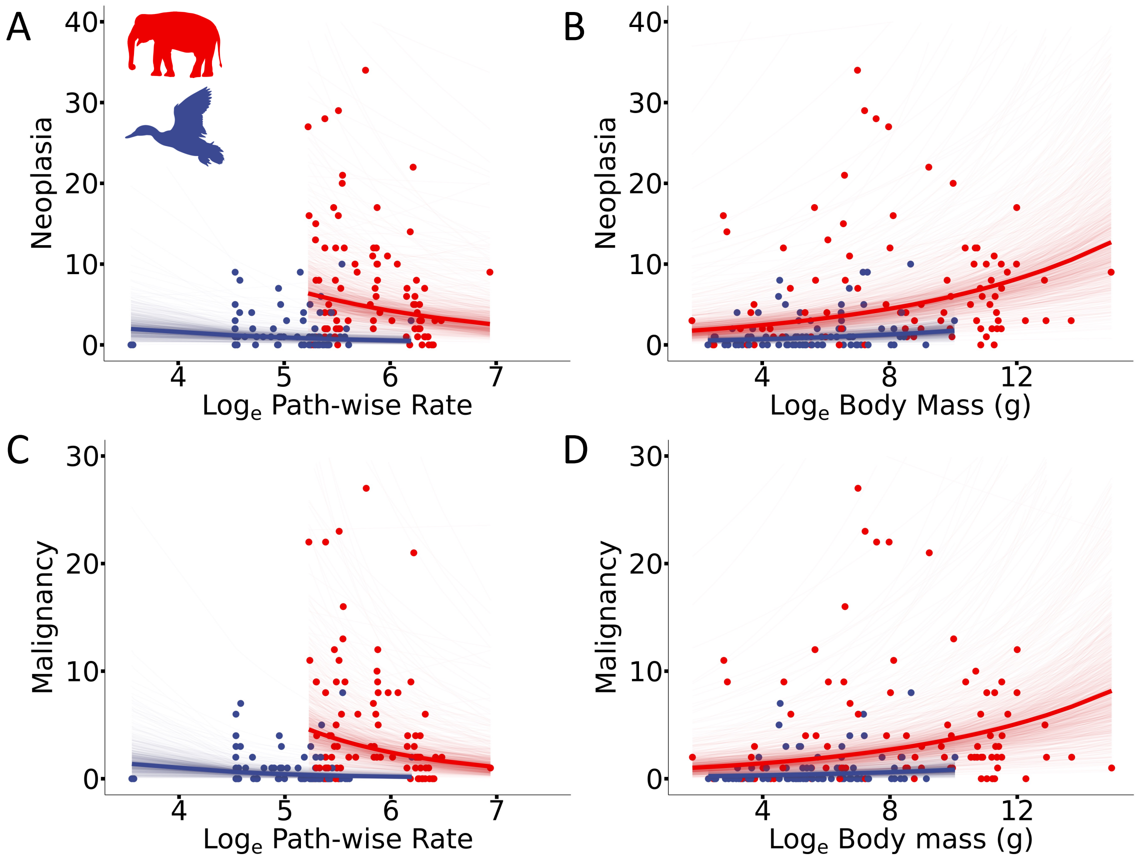

Fig. S14. A negative association between neoplasia or malignancy and path-wise rate in birds and mammals with data points included. In all cases, the posterior predicted slopes are plotted, and the mean average predicted slopes are highlighted. (**A**) Neoplasia prevalence is negatively associated with path-wise rate (see Methods) in birds (blue) and mammals (red) (P_x = 0.027) but (**B**) positively associated with body mass in the same model (P_x = 0). Likewise, (**C**) malignancy is negatively associated with path-wise rate in birds and mammals (P_x = 0.01) but (**D**) positively associated with body mass (P_x = 0.001). Due to the number of species with zero prevalence of Neoplasia or Malignancy the data and fitted model are shown on a non-log transformed Y-axis, hence the non-linearity. The parameters from the fitted model are estimated for regression models that included multiple fixed effects and phylogeny as a random effect. As a result, caution should be taken before drawing conclusion based upon visual inspection. The same results are presented without data points on the log-link scale in Figure 2.
